# Supplementary material for: Generative learning facilitated discovery of high-entropy ceramic dielectrics for capacitive energy storage
Source: Nat Commun. 2024 Jun 10;15:4940. doi: 10.1038/s41467-024-49170-8 (PMC11164696; doi:10.1038/s41467-024-49170-8)
Supplement: Supplementary file 1 — Supplementary Information [file 41467_2024_49170_MOESM1_ESM.pdf]

Supplementary Information

Generative learning facilitated discovery of high-entropy ceramic dielectrics for capacitive energy storage

Wei Li, Zhong-Hui Shen, Run-Lin Liu, Xiao-Xiao Chen, Meng-Fan Guo, Jin-Ming Guo, Hua Hao, Yang Shen, Han-Xing Liu, Long-Qing Chen, Ce-Wen Nan**

Phase-field model

The model size in three-dimensional simulation is 100×100×100 grid points, grid distance is Δx=Δy=Δz=1.0nm, and periodic boundary conditions are adopted. In the phase-field simulation, the electric field was applied along [001] direction, the maximum strength is 1 MV cm^{-1}. The temperature is 300K. The calculation of undoped ceramics is based on the relevant parameters of previous studies.

For $BiFeO_3$,

$a_1=4.9\times10^5$ (T-1103) $C^{-2}m^2N$, $a_{11}=6.5\times10^8$ $C^{-4}m^6N$, $a_{12}=1.0\times10^8$ $C^{-4}m^6N$, $a_{111}=a_{112}=a_{123}=0$;

For $BaTiO_3$,

$a_1=3.34\times10^5$ (T-381) $C^{-2}m^2N$, $a_{11}=4.69\times10^6$ (T-393) -2.02×10^8 $C^{-4}m^6N$, $a_{12}=3.23\times10^8$ $C^{-4}m^6N$, $a_{111}=-5.52\times10^7$ (T-393) + 2.76×10^8 $C^{-6}m^{10}N$, $a_{112}=4.47\times10^9$ C^{-6} m ^{10}N, $a_{123}=4.91\times10^9$ $C^{-6}m^{10}N$;

For $PbTiO_3$,

$a_1=3.8\times10^5$(T-753) $C^{-2}m^2N$, $a_{11}=-7.3\times10^8$ $C^{-4}m^6N$, $a_{12}=7.5\times10^8$ $C^{-4}m^6N$, $a_{111}=2.6\times10^8$ $C^{-6}m^{10}N$, $a_{112}=6.1\times10^8$ $C^{-6}m^{10}N$, $a_{123}=-3.7\times10^{10}$ $C^{-6}m^{10}N$;

$G_{11}/G_{110}=1.2$, $G_{12}/G_{110}=0$, $G_{44}/G_{110}= G'_{44}/G_{110}=0.6$, $G_{110}=7.04\times10^{-11}$ C^{-2} m^4N.

Considered doping effects leads to a decrease in the Curie temperature of these entropy-modulated compositions, we adopted $T_{c2} = T_{c1} - bc$ to describe the Landau coefficient changes. T_{c2} represents the Curie temperature of the material after doping, T_{c1} and b are constants related to the material. The concentration c of doping in different entropy ceramics is listed as follows:

Supplementary Table 1 The parameters of entropy-modulated compositions in phase-field simulations.

	Low-Entropy	Medium-Entropy	Medium-Entropy	High-Entropy
R	0.69	0.90	1.40	1.64
c	0.20	0.25	0.50	0.70

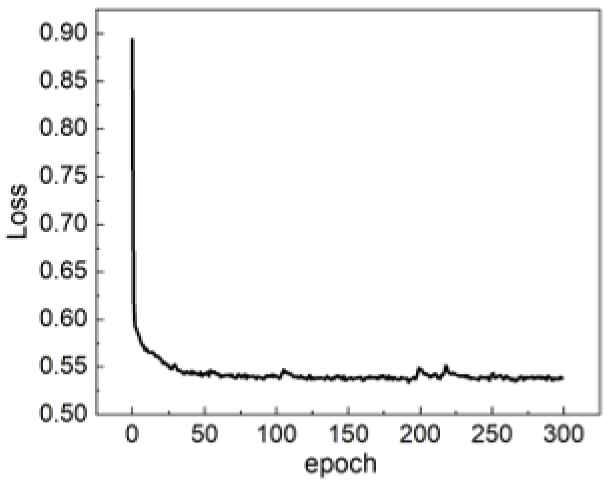

Supplementary Fig. 1 | Reconstruction loss of the encoder-decoder structure. The components of the A and B sites are encoded respectively, with the same structure. The loss function is a weighted sum of cross entropy and maximum mean discrepancy, which levels off as the number of training epoch increases.

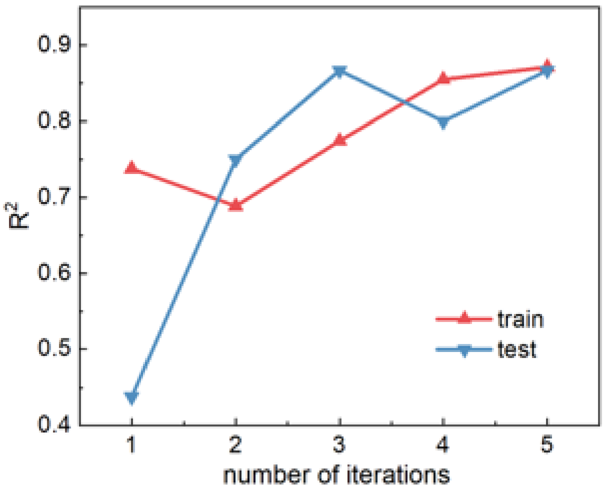

Supplementary Fig. 2 | The results of five-fold cross-validation using a simple ANN classifier. This classifier is divided by an energy density threshold of 65 J cm^{-3}. The batch size is 16 and the learning rate is 5×10^{-3}.

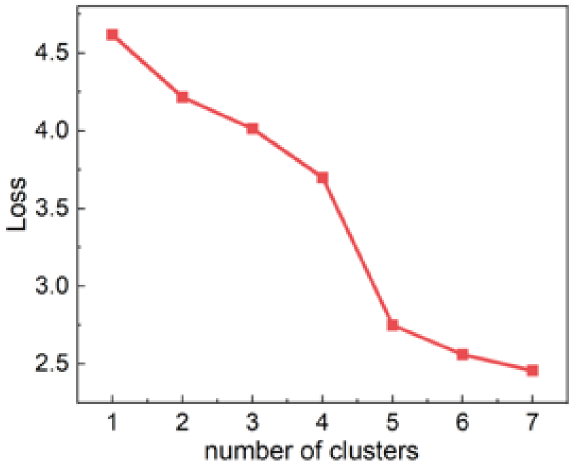

Supplementary Fig. 3 | The optimal number of clusters obtained when computing probability densities for Gaussian mixture models.

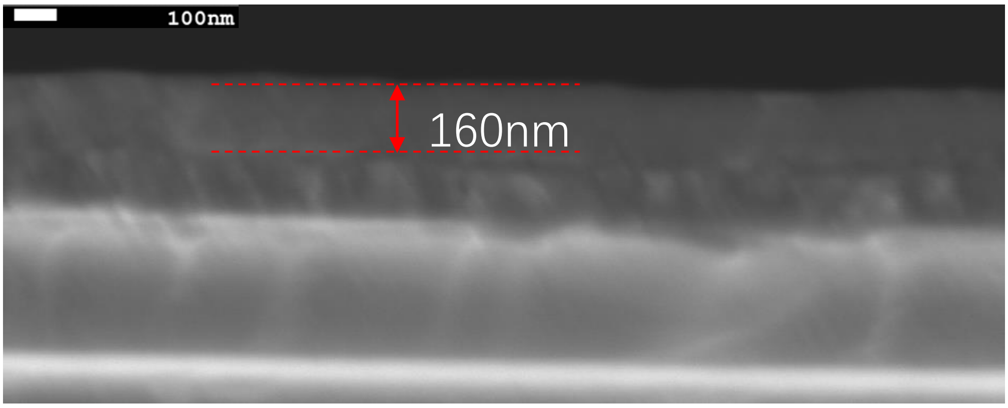

Supplementary Fig. 4 | Cross-sectional SEM image of the C-3 film.

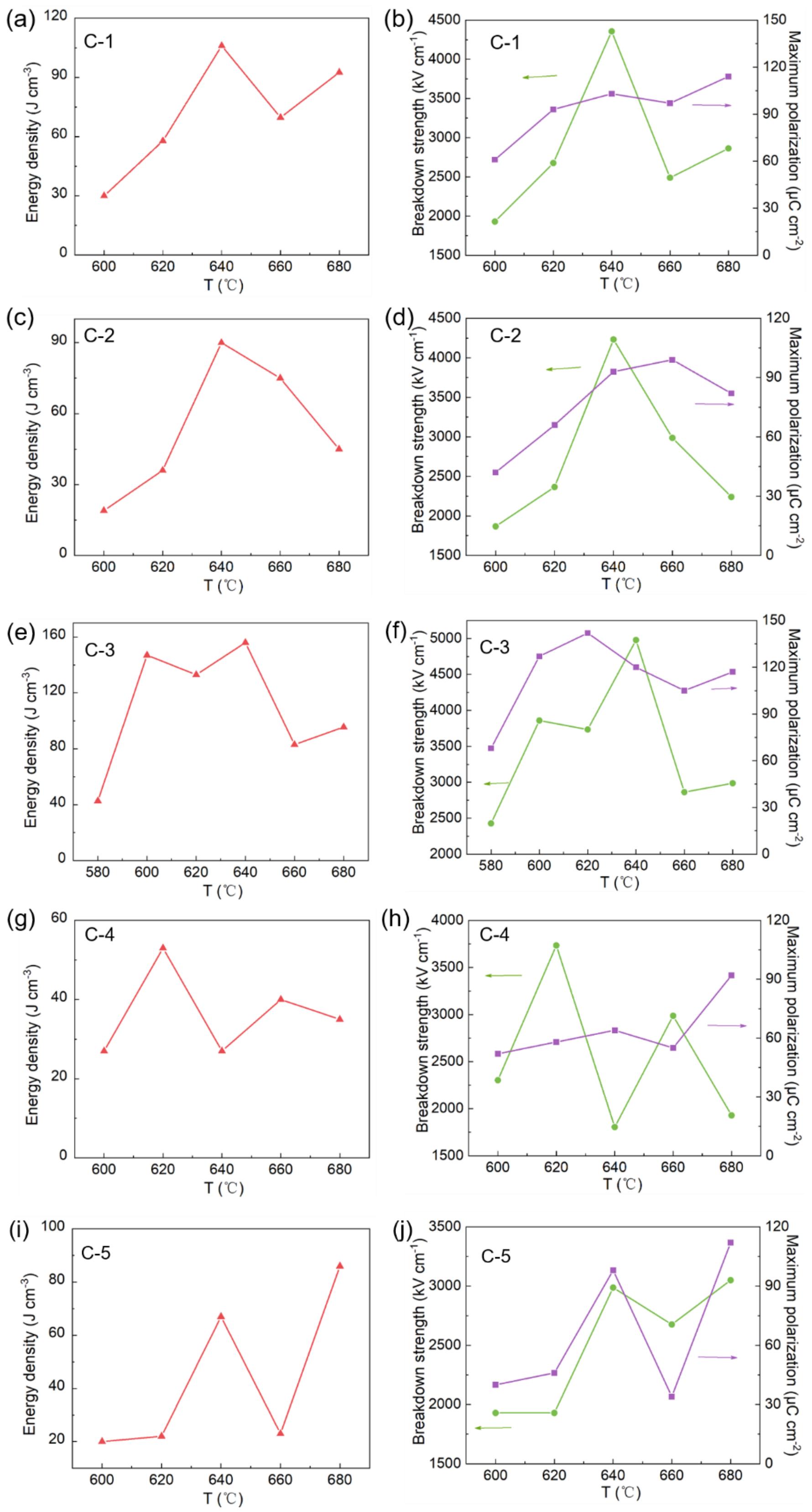

Supplementary Fig. 5 | Energy densities, breakdown strengths and maximum polarizations as a function of temperature for (a, b) C-1 film, (c, d) C-2 film, (e, f) C-3 film, (g, h) C-4 film, (i, j) C-5 film.

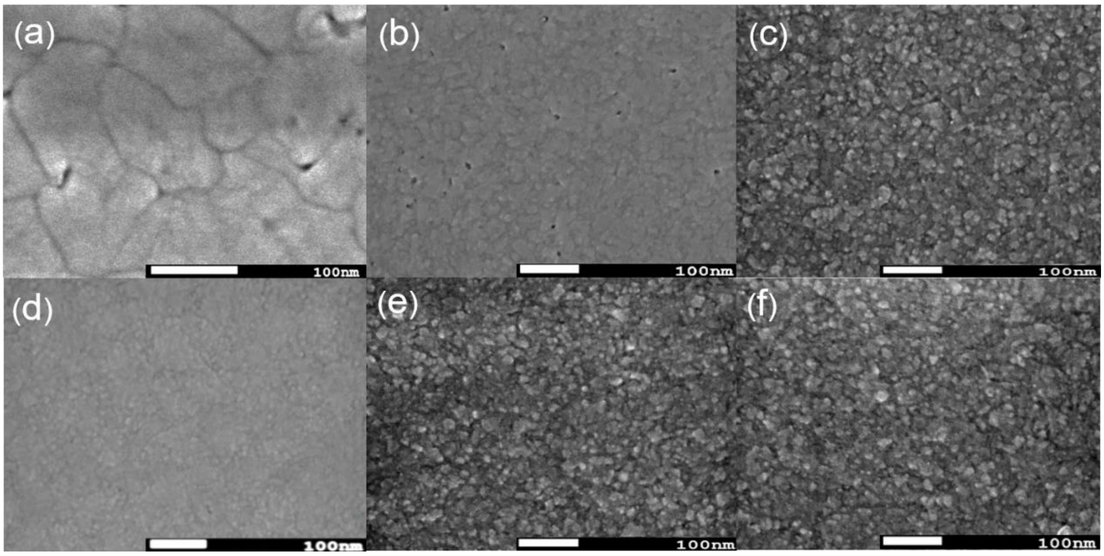

Supplementary Fig. 6 | SEM images of the films of (a) BMT and (b)-(f) C-n (n=1,2,3,4,5).

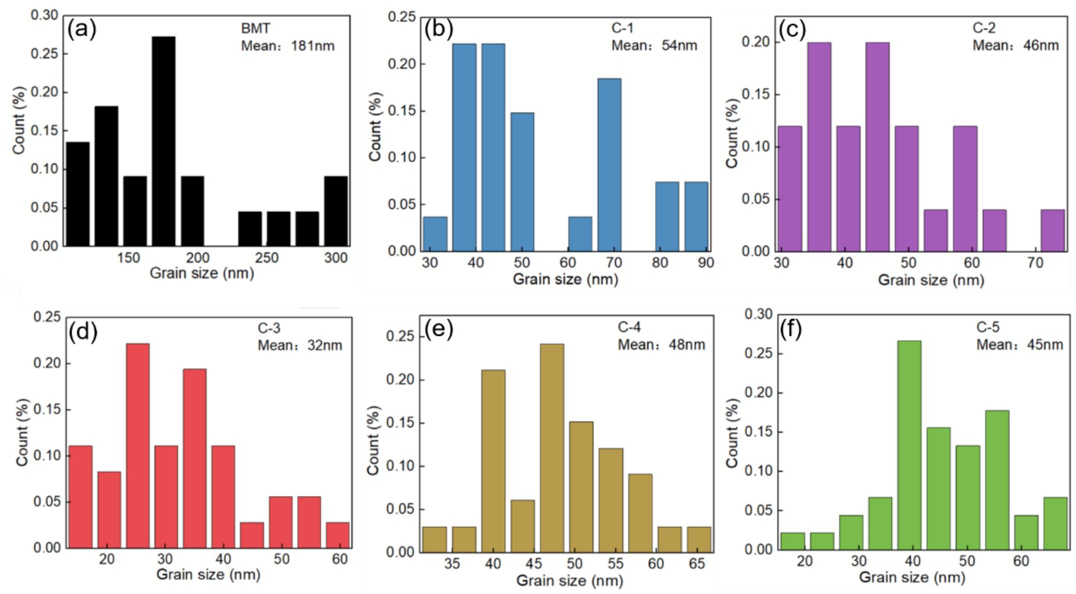

Supplementary Fig. 7 | Grain size distribution of the films of (a) BMT and (b)-(f) C-n (n=1,2,3,4,5).

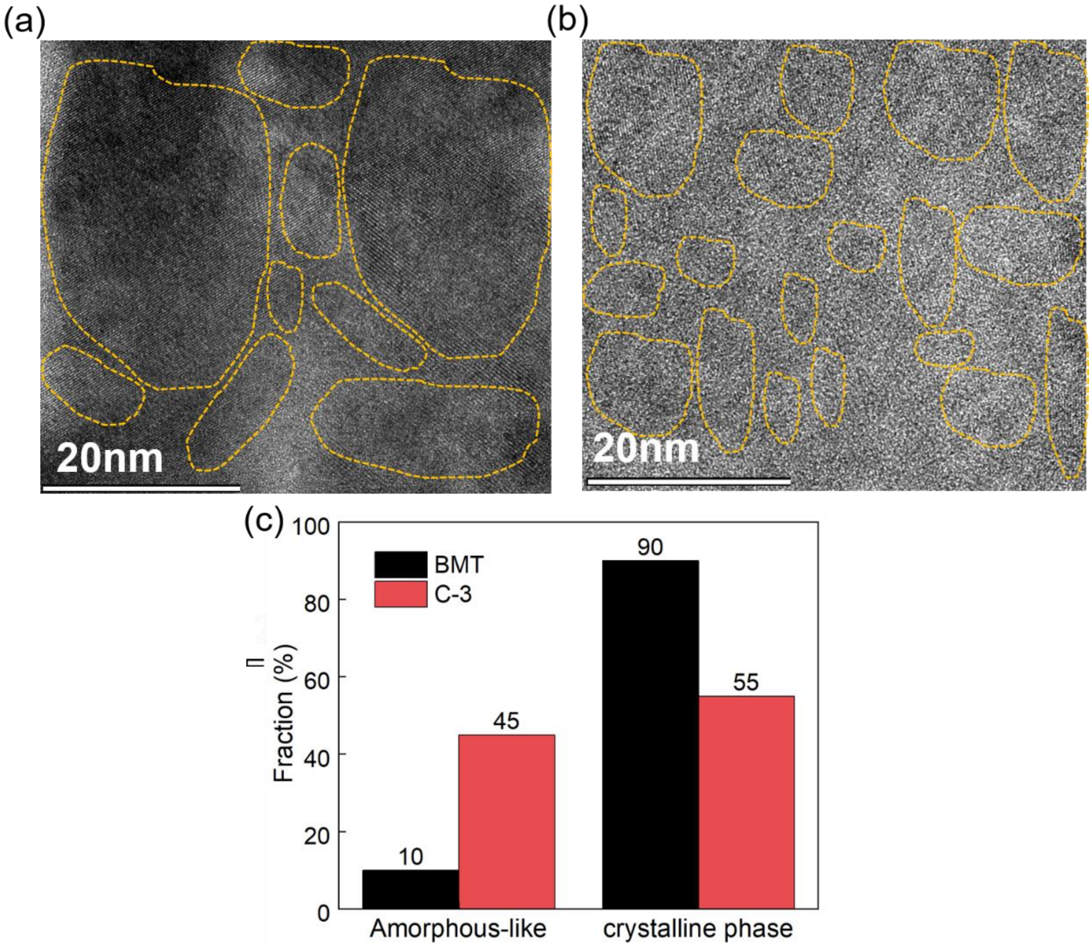

Supplementary Fig. 8 | HR-TEM images of lattice fringes (dotted yellow area) with different directions in (a) BMT and (b) C-3. (c) Fractions of the local crystalline and amorphous-like phases in the samples of BMT and C-3.

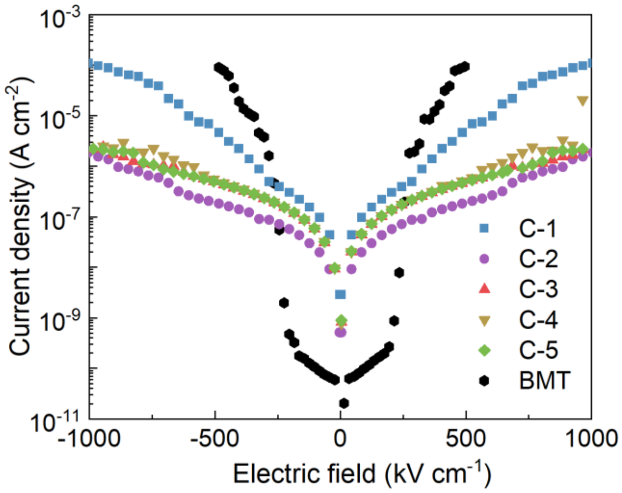

Supplementary Fig. 9 | The I-V curves of BMT and C-n (n=1,2,3,4,5) films.

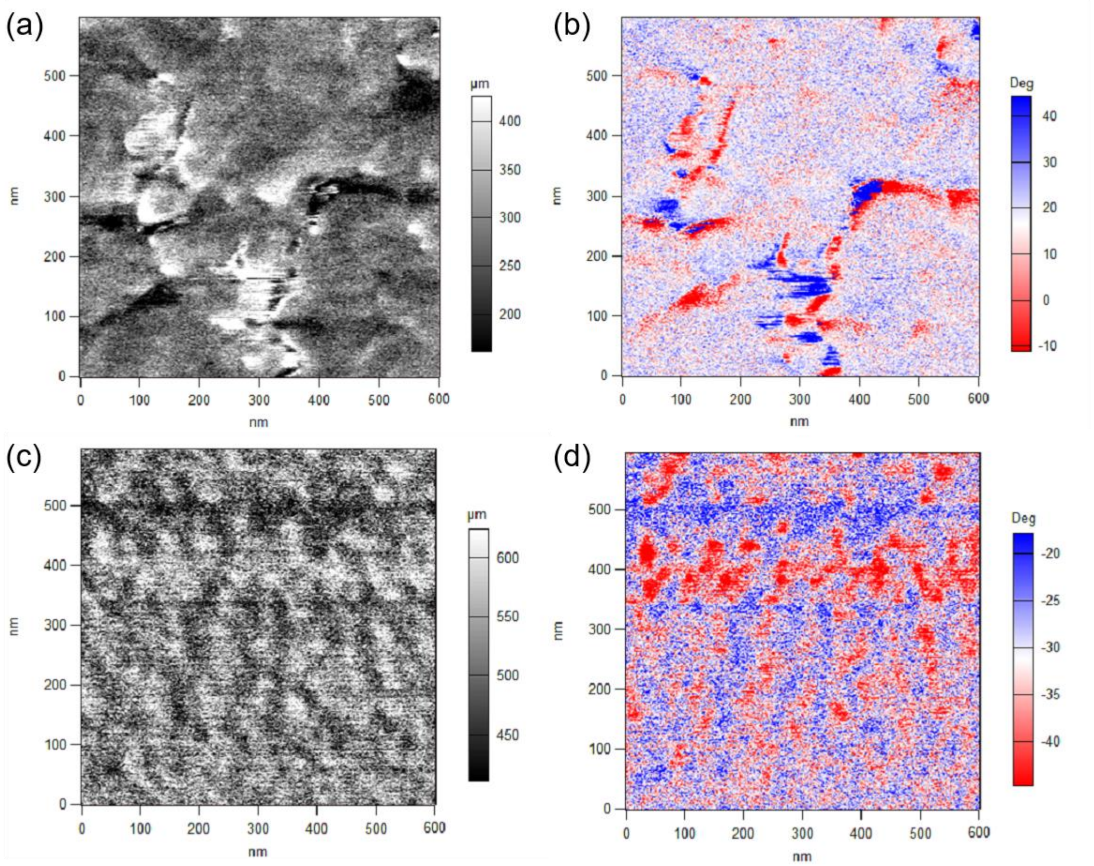

Supplementary Fig. 10 | Lateral PFM amplitude and phase images of (a,b) BMT and (c,d) the C-3 film.

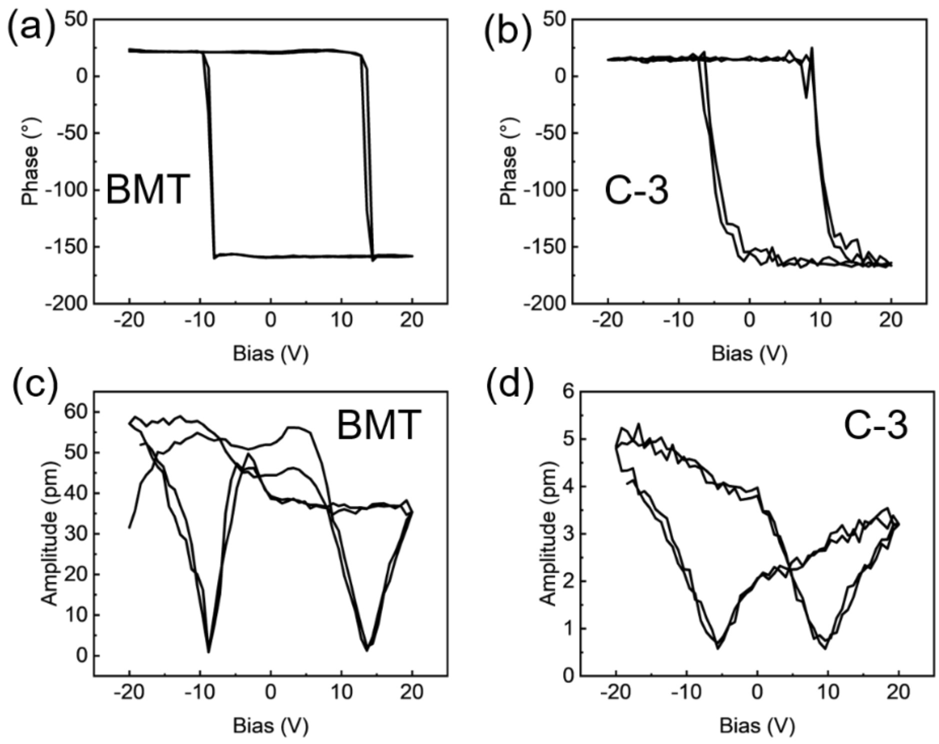

Supplementary Fig. 11 | PFM phase (top panel) and amplitude (bottom panel) of (a,c) BMT thin film and (b,d) C-3 thin film.

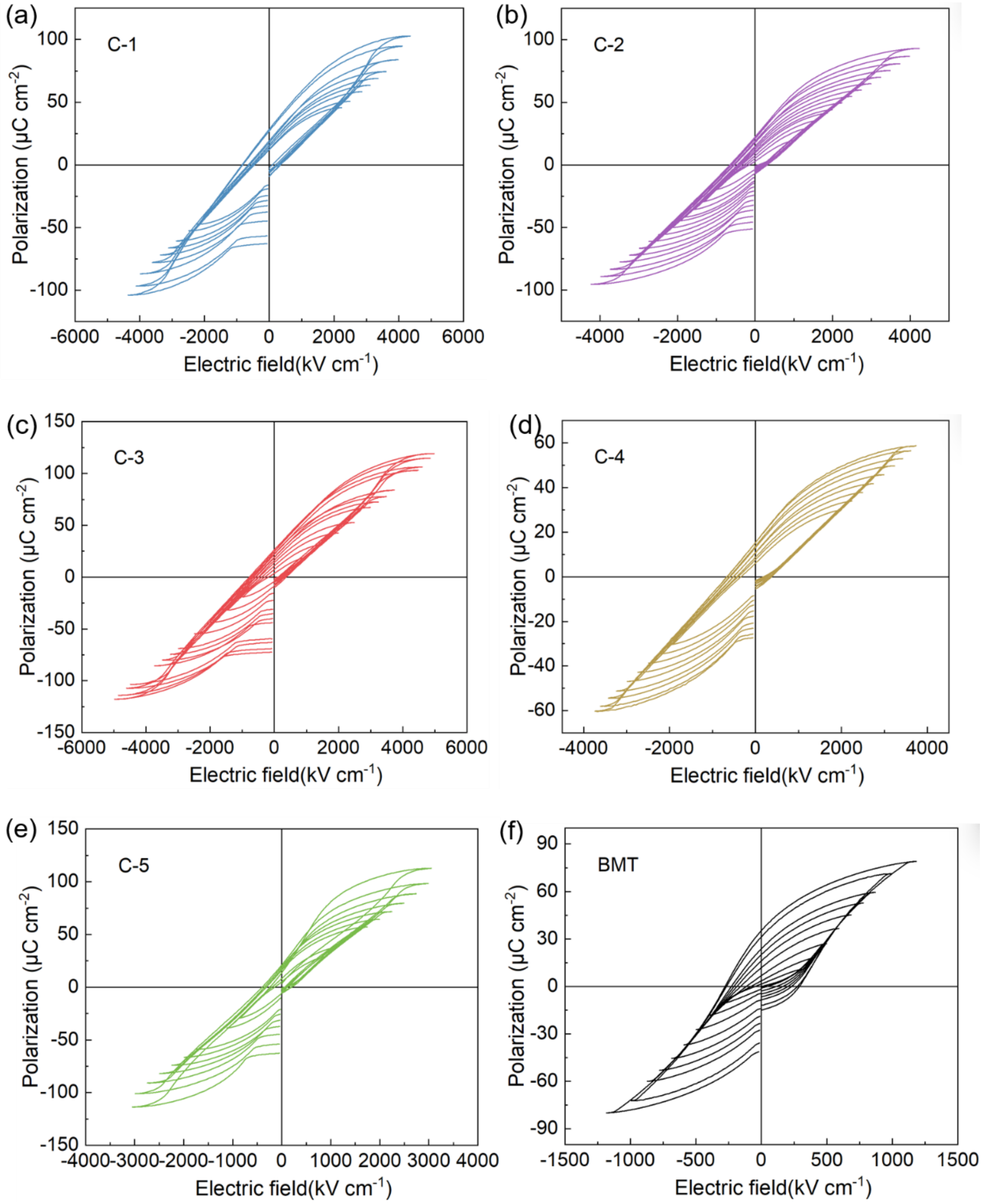

Supplementary Fig. 12 | Bipolar *P*-*E* loops of the films at the electric fields up to their breakdown strength at 1 kHz. (a)-(e), C-n (n=1,2,3,4,5). (f), BMT.

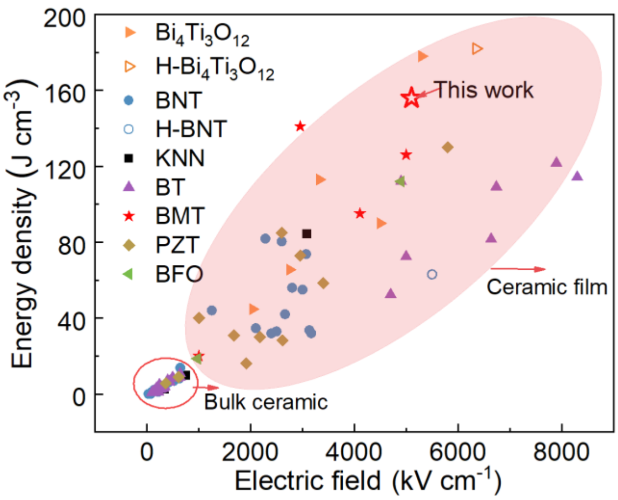

Supplementary Fig. 13 | Comparison of energy storage properties of C-3 high-entropy ceramic film with reported experimental results, where hollow centres are high-entropy data points and solid centres are non-high-entropy data points.

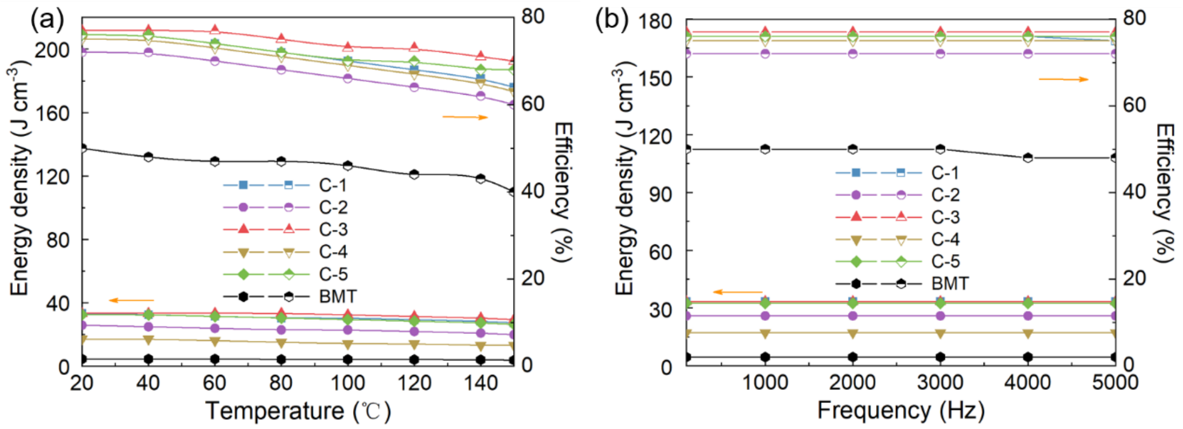

Supplementary Fig. 14 | The temperature dependence (a) and frequency dependence (b) of C-n (n=1,2,3,4,5) films at an electric field of 2000 kV cm^{-1} and BMT is at 1000 kV cm^{-1}.

Supplementary Table 2 77 sets of original data

Composition	U_e (J cm^{-3})	T (°C)
$BiTi_{0.475}Mg_{0.475}Zr_{0.05}O_3$	20.3	600
$BiTi_{0.45}Mg_{0.45}Zr_{0.1}O_3$	16.47	550
	12.24	600
$BiTi_{0.425}Mg_{0.425}Zr_{0.15}O_3$	5.61	600
$BiTi_{0.475}Mg_{0.475}Mn_{0.05}O_3$	30.83	600
$BiTi_{0.45}Mg_{0.45}Mn_{0.1}O_3$	15.63	550
	16.94	600
$BiTi_{0.425}Mg_{0.425}Mn_{0.15}O_3$	55.92	600
$Bi_{0.95}La_{0.05}Ti_{0.5}Mg_{0.5}O_3$	34.97	600
$Bi_{0.9}La_{0.1}Ti_{0.5}Mg_{0.5}O_3$	30.24	550
	21.6	600
$Bi_{0.85}La_{0.15}Ti_{0.5}Mg_{0.5}O_3$	37.53	600
$Bi_{0.95}Sr_{0.05}Ti_{0.5}Mg_{0.5}O_3$	19.43	600
$Bi_{0.9}Sr_{0.1}Ti_{0.5}Mg_{0.5}O_3$	15.94	600
$Bi_{0.85}Sr_{0.15}Ti_{0.5}Mg_{0.5}O_3$	14.26	600
$Bi_{0.9}La_{0.1}Ti_{0.45}Mg_{0.45}Mn_{0.05}Zr_{0.05}O_3$	7.14	500
	25.32	550
	53.83	600
	48.83	650
	55.29	700
$Bi_{0.9}La_{0.1}Ti_{0.4}Mg_{0.4}Mn_{0.1}Zr_{0.1}O_3$	12.12	500
	46.94	550
	57.33	600
	42.42	650
	44.27	700
$Bi_{0.9}La_{0.1}Ti_{0.35}Mg_{0.35}Mn_{0.15}Zr_{0.15}O_3$	21.22	500
	25.45	550
	34.57	600
	42.91	650
$Bi_{0.9}La_{0.1}Ti_{0.3}Mg_{0.3}Mn_{0.2}Zr_{0.2}O_3$	19.52	500
	26.11	550
	36.21	600
	11.58	650
$Bi_{0.9}La_{0.1}Ti_{0.25}Mg_{0.25}Mn_{0.25}Zr_{0.25}O_3$	19.08	500
	16.33	550
	8.85	600
	3.4	650
$Bi_{0.9}La_{0.1}Ti_{0.4}Mg_{0.4}Zr_{0.1}Hf_{0.1}O_3$	33.74	600
	21.92	650
	10.21	700
$Bi_{0.9}La_{0.1}Ti_{0.7}Mg_{0.1}Zr_{0.1}Hf_{0.1}O_3$	32.65	600
	23.97	650

	23.52	700
$Bi_{0.9}La_{0.1}Ti_{0.61}Mg_{0.13}Zr_{0.13}Hf_{0.13}O_3$	13.51	600
	15.68	650
	26.25	700
$Bi_{0.9}La_{0.1}Sr_{0.05}Ti_{0.4}Mg_{0.4}Mn_{0.1}Zr_{0.1}O_3$	40.66	550
	50.13	600
	37.93	650
$Bi_{0.9}La_{0.1}Sr_{0.1}Ti_{0.4}Mg_{0.4}Mn_{0.1}Zr_{0.1}O_3$	12.09	550
	31.41	600
	29.92	650
$Bi_{0.94}La_{0.03}Sr_{0.03}Ti_{0.4}Mg_{0.4}Mn_{0.1}Zr_{0.1}O_3$	28.14	600
	38.06	620
$Bi_{0.94}La_{0.03}Sr_{0.03}Ti_{0.35}Mg_{0.35}Mn_{0.15}Zr_{0.15}O_3$	21.78	600
	12.82	620
$Bi_{0.92}La_{0.04}Sr_{0.04}Ti_{0.4}Mg_{0.4}Mn_{0.1}Zr_{0.1}O_3$	17.91	600
	36.37	620
$Bi_{0.92}La_{0.04}Sr_{0.04}Ti_{0.35}Mg_{0.35}Mn_{0.15}Zr_{0.15}O_3$	14.95	600
	27.13	620
$Bi_{0.9}La_{0.05}Sr_{0.05}Ti_{0.4}Mg_{0.4}Mn_{0.1}Zr_{0.1}O_3$	87.29	600
	74.42	650
	62	700
$Bi_{0.9}La_{0.05}Sr_{0.05}Ti_{0.4}Mg_{0.35}Mn_{0.35}Zr_{0.15}O_3$	22.24	600
	54.58	650
	36.7	700
$Bi_{0.9}La_{0.05}Sr_{0.05}Ti_{0.4}Mg_{0.4}Zr_{0.1}Hf_{0.1}O_3$	41.95	600
	36.97	620
	15.39	650
$Bi_{0.9}La_{0.05}Ca_{0.05}Ti_{0.4}Mg_{0.4}Zr_{0.1}Mn_{0.1}O_3$	23.58	600
	36.45	650
$Bi_{0.9}La_{0.05}Sr_{0.05}Ti_{0.4}Mg_{0.4}Mn_{0.1}Hf_{0.1}O_3$	53.82	600
	46.6	620
	38	650
$Bi_{0.9}La_{0.05}Sr_{0.05}Ti_{0.4}Mg_{0.4}Mn_{0.1}Zr_{0.1}Hf_{0.1}O_3$	22.91	600
	13.12	620
	25.93	650

T: annealing temperature

Supplementary Table 3 Original data of the first 10 rows.

Bi	La	Sr	Ca	Ti	Mg	Mn	Zr	Hf
0.90	0.10	0.0	0.0	0.45	0.45	0.05	0.05	0.0
0.90	0.10	0.0	0.0	0.40	0.40	0.10	0.10	0.0
0.90	0.10	0.0	0.0	0.35	0.35	0.15	0.15	0.0
0.90	0.10	0.0	0.0	0.30	0.30	0.20	0.20	0.0
0.90	0.10	0.0	0.0	0.25	0.25	0.25	0.25	0.0
0.90	0.05	0.05	0.0	0.40	0.40	0.10	0.10	0.0
0.94	0.03	0.03	0.0	0.40	0.40	0.10	0.10	0.0
0.94	0.03	0.03	0.0	0.35	0.35	0.15	0.15	0.0
0.9	0.10	0.00	0.0	0.70	0.10	0.00	0.10	0.1
0.95	0.00	0.05	0.0	0.50	0.50	0.00	0.00	0.0

Supplementary Table 4 Reconstruction data of the first 10 rows.

Bi	La	Sr	Ca	Ti	Mg	Mn	Zr	Hf
0.8980	0.1020	0.0000	0.0000	0.4532	0.4472	0.0458	0.0538	0.0000
0.8980	0.1020	0.0000	0.0000	0.4011	0.3964	0.1008	0.1017	0.0000
0.8980	0.1020	0.0000	0.0000	0.3509	0.3447	0.1548	0.1497	0.0000
0.8980	0.1020	0.0000	0.0000	0.2978	0.2941	0.2079	0.2002	0.0001
0.8980	0.1020	0.0000	0.0000	0.2471	0.2449	0.2583	0.2493	0.0004
0.8986	0.0476	0.0537	0.0000	0.4011	0.3964	0.1008	0.1017	0.0000
0.9404	0.0270	0.0325	0.0001	0.4011	0.3964	0.1008	0.1017	0.0000
0.9404	0.0270	0.0325	0.0001	0.3509	0.3447	0.1548	0.1497	0.0000
0.8980	0.1020	0.0000	0.0000	0.6907	0.0964	0.0000	0.1074	0.1054
0.9524	0.0003	0.0473	0.0000	0.4971	0.5020	0.0007	0.0000	0.0002

Supplementary Table 5 Physical descriptors in the machine learning model.

Descriptor	Explanation
R_A(Å)	Ionic radii of A-site (12-coordination)
R_B(Å)	Ionic radii of B-site (12-coordination)
AR_A(Å)	Atomic radius of A-site element
AR_B(Å)	Atomic radius of B-site element
A-O_A(Å)	Ideal A–O bond distance
A-O_B(Å)	Ideal B–O bond distance
V_A	Atomic volume of A-site element
V_B	Atomic volume of B-site element
VWR_A	Crystallographic van der Waals radii of A-site element
VWR_B	Crystallographic van der Waals radii of B-site element
PE_A	Period of A-site element in element period table
PE_B	Period of B-site element in element period table
W_A	Relative atomic mass of A-site element
W_B	Relative atomic mass of B-site element
AN_A	Atomic number of A-site element in element period table
AN_B	Atomic number of B-site element in element period table
EN_A	A-site electronegativity (Pearson 1988)
EN_B	B-site electronegativity
EI_A	First energy ionization of A-site element
EI_B	First energy ionization of B-site element
EA_A	Electron affinity of A-site element
EA_B	Electron affinity of B-site element
t	Tolerance factor calculated by ionic radii
μ	Octahedral factor calculated by ionic radii
**E**	Entropy calculated by compositions

Supplementary Table 6 Compositions and U_e of the HEDs designed in this work**.**

HED	Title	True U_e	Entropy	Uncertainty	Rank
$Bi_{0.87}La_{0.1}Sr_{0.03}Ti_{0.42}Mg_{0.4}Mn_{0.14}Zr_{0.04}O_3$	C-1	106	1.59R	6.5	1
$Bi_{0.9}La_{0.05}Sr_{0.05}Ti_{0.4}Mg_{0.39}Mn_{0.13}Zr_{0.08}O_3$	C-2	90	1.59R	4.8	2
$Bi_{0.87}La_{0.08}Sr_{0.05}Ti_{0.41}Mg_{0.39}Mn_{0.15}Zr_{0.05}O_3$	C-3	156	1.64R	10.1	3
$Bi_{0.9}La_{0.05}Sr_{0.05}Ti_{0.40}Mg_{0.30}Mn_{0.13}Zr_{0.07}O_3$	C-4	53	1.57R	6.7	4
$Bi_{0.89}La_{0.06}Sr_{0.05}Ti_{0.41}Mg_{0.39}Mn_{0.12}Zr_{0.08}O_3$	C-5	86	1.61R	11.2	5
