# Supplementary material for: Generative learning facilitated discovery of high-entropy ceramic dielectrics for capacitive energy storage
Source: Nat Commun. 2024 Jun 10;15:4940. doi: 10.1038/s41467-024-49170-8 (PMC11164696; doi:10.1038/s41467-024-49170-8)
Supplement: Supplementary file 3 — Reporting Summary [file 41467_2024_49170_MOESM3_ESM.pdf]

nature portfolio

Corresponding author(s): Zhong-Hui Shen, Ce-Wen Nan

Last updated by author(s): May 6, 2024

Reporting Summary

Nature Portfolio wishes to improve the reproducibility of the work that we publish. This form provides structure for consistency and transparency in reporting. For further information on Nature Portfolio policies, see our Editorial Policies and the Editorial Policy Checklist.

Statistics

For all statistical analyses, confirm that the following items are present in the figure legend, table legend, main text, or Methods section.

n/a	Confirmed	
☐	☒	The exact sample size (*n*) for each experimental group/condition, given as a discrete number and unit of measurement
☐	☒	A statement on whether measurements were taken from distinct samples or whether the same sample was measured repeatedly
☐	☒	The statistical test(s) used AND whether they are one- or two-sided<br>*Only common tests should be described solely by name; describe more complex techniques in the Methods section.*
☒	☐	A description of all covariates tested
☐	☒	A description of any assumptions or corrections, such as tests of normality and adjustment for multiple comparisons
☐	☒	A full description of the statistical parameters including central tendency (e.g. means) or other basic estimates (e.g. regression coefficient) AND variation (e.g. standard deviation) or associated estimates of uncertainty (e.g. confidence intervals)
☐	☒	For null hypothesis testing, the test statistic (e.g. F, t, r) with confidence intervals, effect sizes, degrees of freedom and P value noted<br>*Give P values as exact values whenever suitable.*
☐	☒	For Bayesian analysis, information on the choice of priors and Markov chain Monte Carlo settings
☐	☒	For hierarchical and complex designs, identification of the appropriate level for tests and full reporting of outcomes
☐	☒	Estimates of effect sizes (e.g. Cohen's *d*, Pearson's *r*), indicating how they were calculated

Our web collection on statistics for biologists contains articles on many of the points above.

Software and code

Policy information about availability of computer code

Data collection	All data are collected from experiments and no software was used in this process.
Data analysis	The code used to data analysis are deposited at https://github.com/lowwo/high-entropy-dielectrics-design.

For manuscripts utilizing custom algorithms or software that are central to the research but not yet described in published literature, software must be made available to editors and reviewers. We strongly encourage code deposition in a community repository (e.g. GitHub). See the Nature Portfolio guidelines for submitting code & software for further information.

Data

Policy information about availability of data

All manuscripts must include a data availability statement. This statement should provide the following information, where applicable:

- Accession codes, unique identifiers, or web links for publicly available datasets
- A description of any restrictions on data availability
- For clinical datasets or third party data, please ensure that the statement adheres to our policy

All data used are available within this paper and Supplementary Information. Further information can be acquired from the corresponding authors upon reasonable request.

Research involving human participants, their data, or biological material

Policy information about studies with human participants or human data. See also policy information about sex, gender (identity/presentation), and sexual orientation and race, ethnicity and racism.

Reporting on sex and gender	No sex and gender are used in this work.
Reporting on race, ethnicity, or other socially relevant groupings	We don't use the socially constructed or socially relevant categorization variables in our manuscript.
Population characteristics	The participants in this work are graduate students and professors, who ages range from 20s to 60s. No other special population characteristics are needed to stated.
Recruitment	The participants in this work are graduate students and professors, and all material preparation and performance analysis are based on the results of instrument testing and characterization.
Ethics oversight	This work is carried out in Wuhan University of Technology.

Note that full information on the approval of the study protocol must also be provided in the manuscript.

Field-specific reporting

Please select the one below that is the best fit for your research. If you are not sure, read the appropriate sections before making your selection.

☐ Life sciences ☐ Behavioural & social sciences ☒ Ecological, evolutionary & environmental sciences

For a reference copy of the document with all sections, see nature.com/documents/nr-reporting-summary-flat.pdf

Ecological, evolutionary & environmental sciences study design

All studies must disclose on these points even when the disclosure is negative.

Study description	This work is carried out to find high-performance high-entropy dielectrics by machine learning based on 77 experimental results. Then, five directed experiments are performed to verify the theoretical predictions by measuring the energy density and efficiency.
Research sample	The material samples in this work are generated by designing 77 groups of experiments as the initial input data. Then, generative learning-based model is used to generate more data and screen high potential components.
Sampling strategy	The initial experimental points in this work is based on the randomness and the distribution of elements, and the stability of the machine learning model. Firstly, these 77 data sets are random variations that we have designed based on the gradual increase in the type and number of elements, with some compositions designed on the base of experience and the results of existing experiments that have been carried out. Secondly, these variations cover every element as much as possible because of the large number of element types and the possibility of changes in elemental content. In addition, those 77 initial data could successfully meet the encoder-decoder model to reconstruct the components.
Data collection	The performance of initial 77 experimental samples are measured by ferroelectric testing instrument. Then, the author of Wei Li calculates corresponding energy density and efficiency to form 77 sets of data to train the machine learning model.
Timing and spatial scale	The collection of data is done by the same device in one month, and its performance hardly changes due to the stability of the ceramic materials.
Data exclusions	No data is excluded from the analyses.
Reproducibility	For each sample, we performed at least eight repeatable tests to assess material properties.
Randomization	All samples are used in our study and there is no grouping.
Blinding	To generate suitable initial data to train the machine learning model, we prepare 77 sets of experiments according to different element distribution in high-entropy ceramic dielectrics. So blinding is not relevant to out study.

Did the study involve field work? ☐ Yes ☒ No

Reporting for specific materials, systems and methods

We require information from authors about some types of materials, experimental systems and methods used in many studies. Here, indicate whether each material, system or method listed is relevant to your study. If you are not sure if a list item applies to your research, read the appropriate section before selecting a response.

Materials & experimental systems

n/a	Involved in the study
☒	☐ Antibodies
☒	☐ Eukaryotic cell lines
☒	☐ Palaeontology and archaeology
☒	☐ Animals and other organisms
☒	☐ Clinical data
☒	☐ Dual use research of concern
☒	☐ Plants

Methods

n/a	Involved in the study
☒	☐ ChIP-seq
☒	☐ Flow cytometry
☒	☐ MRI-based neuroimaging

Plants

Seed stocks	No seed stocks and other plant material are used in this work.
Novel plant genotypes	No novel plant genotypes were generated in this work.
Authentication	No authentication procedures were used in this work.
